# Supplementary material for: Vitamin D and the Ability to Produce 1,25(OH)2D Are Critical for Protection from Viral Infection of the Lungs
Source: Nutrients. 2022 Jul 26;14(15):3061. doi: 10.3390/nu14153061 (PMC9332570; doi:10.3390/nu14153061)
Supplement: Supplementary file 1 [file nutrients-14-03061-s001.zip › nutrients-1817784-supplementary.pdf]

Supplementary Table S1. Primer sequences for qPCR.

**Target gene**	**Forward Primer (5’-3’)**	**Reverse Primer (5’-3’)**
Vdr	5’-CTG CAC CTC CTC ATC TGT GA-3’	5’-CCC CTT CAA TGG AGA TTG C-3’
Cyp24A1	5’-ACC CCC AAG GTC CGT GAC ATC-3	5’-CCA GTT GGG TCC AGG TAA GG-3’
Cyp27B1	5′-CCG CGG GCT ATG CTG GAA C-3′	5′-CTC TGG GCA AAG GCA AAC ATC TGA-3′
Ifnα	5'-GGA CTT TGG ATT CCC GCA GGA GAA G-3'	5'- GCT GCA TCA GAC AGC CTT GCA GGT C-3'
Ifnβ	5’-AGG GCG GAC TTC AAG ATC-3’	5’-CTC ATT CCA CCC AGT GCT-3’
Ifnγ	5’-TGC ATC TTG GCT TGG CAG CTC TTC-3’	5’-GGG TTG ACC TCA AAC TTG GCA-3’
H1N1 M gene	5’-AGA TGA GTC TTC TAA CCG AGG TCG-3’	5’-TCG AGA TCG GTG TTC TTT CC-3’

Supplementary Table S2. Histological evaluation of SARS-CoV-2 infected mouse.

**Parameter**	**Score = 0**	**Score = 1**	**Score = 2**	**Score = 3**
**Perivascular Infiltrates (0-3)**	None	Up to 10% vessels affected	Up to 25% vessels affected	≥ 25% vessels affected
**Lymphocyte dominant (0-1)**	No	Yes		
**Endothelial reactivity (0-2)**	None	Any/multi-focal	Generalized	
**Other vascular parameters (0-1)**	Absent	Present		
**Type II pneumocyte hypertrophy (0-1)**	Absent	Present		
**Pneumonia extent (0-3)**	None	Minimal, focal	Minimal, multi-focal	Mild, multi-focal
**Alveolar remodeling (0-1)**	None	Any		
**Interstitial pneumonia (0-3)**	None	Up to 10% affected	10- 25% affected	≥ 25% affected
**Intra-alveolar inflammation (0-3)**	None	Up to 10% affected	Up to 25% affected	≥ 25% affected

[1]Histopathology criteria for scoring lung sections of SARS-CoV-2 infected mice.

Supplementary Table S3. Histological evaluation of SARS-CoV-2 infected.

Parameter	Score = 0	Score = 1	Score = 2	Score = 3	Score = 4
**Lesions (0-4)**	None	Up to 25% affected	25-50% affected	50-75% affected	> 75% affected
**Alveoli (0-3)**	None	Mild edema and/or infiltrate	Moderate to severe edema or infiltrate	Diffuse alveolar damage	N/A
**Bronchioles (0-3)**	None	Mild peribronchiolar cuffing and/or infiltrate, or epithelial degeneration	Moderate peribronchiolar cuffing and/or infiltrate with epithelial degeneration	Severe peribronchiolar cuffing and/or infiltrate with epithelial necrosis	N/A
**Blood vessels (0-3)**	None	Perivascular edema, mild perivascular cuffing	Perivascular cuffing in >30% of vessels, minimal vasculitis	Vasculitis with thrombosis	N/A
**Hemorrhage (0-2)**	None	Mild	Severe	N/A	N/A
**Type II pneumocyte hyperplasia (0-2)**	None	Early change, mild	Marked change, widespread	N/A	N/A

[1]Histopathology criteria for scoring lung sections of SARS-CoV-2 infected hamsters.

Supplementary Table S4. Histological evaluation of H1N1 infected.

Parameter	Score = 0	Score = 1	Score = 2	Score = 3
**Total lymphocyte infiltration (0-3)**	None	Some inflammation	< 3 foci of inflammation	> 3 foci of inflammation
**Perivascular infiltration (0-3)**	None	Some lymphocytes around bronchiole or blood vessel	Lymphoid aggregates around bronchioles or blood vessel	Multiple lymphoid aggregates
**Alveolar Hemorrhage (0-3)**	None	Few hemorrhagic lesions	Multiple lesions	Hemorrhage with alveolar damage

[1]Histopathology criteria for scoring lung sections of H1N1 influenza infected mice.
